# Biotic Versus Abiotic Control of Primary Production Identified in a Common Garden Experiment

**DOI:** 10.1038/s41598-019-48512-7

**Published:** 2019-08-19

**Authors:** Gary E. Belovsky, Jennifer B. Slade

**Affiliations:** 1 Environmental Research Center, University of Notre Dame, Notre Dame, IN 46556 USA; 2 Department of Biological Sciences, University of Notre Dame, Notre Dame, IN 46556 USA

**Keywords:** Community ecology, Ecosystem ecology

## Abstract

Understanding drivers of ecosystem primary production is a foundational question in ecology that grows in importance with anthropogenic stresses (e.g., climate change). Traditionally, ecosystem production is considered to be abiotically controlled at large spatial scales (e.g., precipitation, temperature, etc.), which underlies forecasting climate change impacts. Using a “common garden” experiment over 10 years at two sites with the same plant and grasshopper species, we show that primary production is strongly influenced by biotic factors (herbivory and plant adaptations to it) at finer spatial scales by creating positive feedbacks, which reverse relative productivity of ecosystems expected from abiotic conditions alone. Our results without herbivory indicate that one site has 26% less annual net primary production (ANPP) than the other site. With herbivory, the sites reverse in ANPP, so the site with lower ANPP without herbivory now is 15% greater than the site with higher ANPP without herbivory, as they respectively increase by 6% and decline by 33%. This reversal is due to changing nitrogen availability (N), as N becomes 16% greater at the higher ANPP site with herbivory, respectively a 3% increase and 41% decline in N. The ANPP and N changes are observed, even though the sites are a few kilometers apart and have the same grasshopper and plant species.

## Introduction

Abiotic factors (e.g., precipitation and temperature) have traditionally been viewed as the main drivers of ecosystem primary production^1^, and this has been the principal basis for forecasting climate change effects on ecosystem functioning^2^. However, this forecasting may be misleading if biotic effects are ignored. For example, there is mounting evidence supporting an early claim^3^ that herbivory strongly affects ecosystem primary production by modifying nutrient cycling^4–9^. This biotic influence can create two types of positive feedbacks: (1) herbivory may enhance nutrient cycling and thereby increase primary production, or (2) herbivory may diminish nutrient cycling and thereby decrease primary production. If these biotic positive feedbacks strongly impact nutrient cycling and primary production, they will reduce the impact of abiotic processes^2^ that are traditionally considered all important^1^. Therefore, how important this biotic effect is relative to abiotic effects is crucial for forecasting climate change impacts on ecosystem functioning and needs to be addressed. A productive way to distinguish between the roles played by abiotic and biotic influences is a “common garden” experiment. Starting with a plant/herbivore system that exhibits the alternative positive feedback effects between two sites, the system’s components (soil, plants and herbivores) can be translocated between the sites and the ecosystem functioning (N and ANPP) can be compared with the site of origin.

An appropriate plant/herbivore system for this “common garden” experiment is provided by a grassland (National Bison Range, Montana, USA). Here two sites differing by <30 m in elevation and within <7.5 km of each other have the same plant species (*Pascopyrum smithii* and *Poa pratensis*) and the same most common grasshopper (*Melanoplus sanuinipes*: Orthopteran, Acrididae), but each exhibits one of the alternative positive feedback effects on N-cycling and ANPP^9–12^. Previous experiments indicated that if the grasshopper prefers to feed on the slower decomposing plant (*Pascopyrum smithii*), then N-cycling and ANPP increase (Site A: UTM 713570 E 5248100N), while preferential feeding on the faster decomposing plant (*Poa pratensis*) leads to N-cycling and ANPP decreasing (Site B: UTM 706453 E 5249980N)^9–12^. Soils and grasses from the two sites can be translocated between the two sites creating different independent combinations (experimental ecosystems) to which the grasshopper can be added to assess the effects of microclimate, soil, and plant differences on the previously observed positive feedback effects of herbivory on N-cycling and ANPP.

## Results

### Without grasshoppers

**Figure 1:**
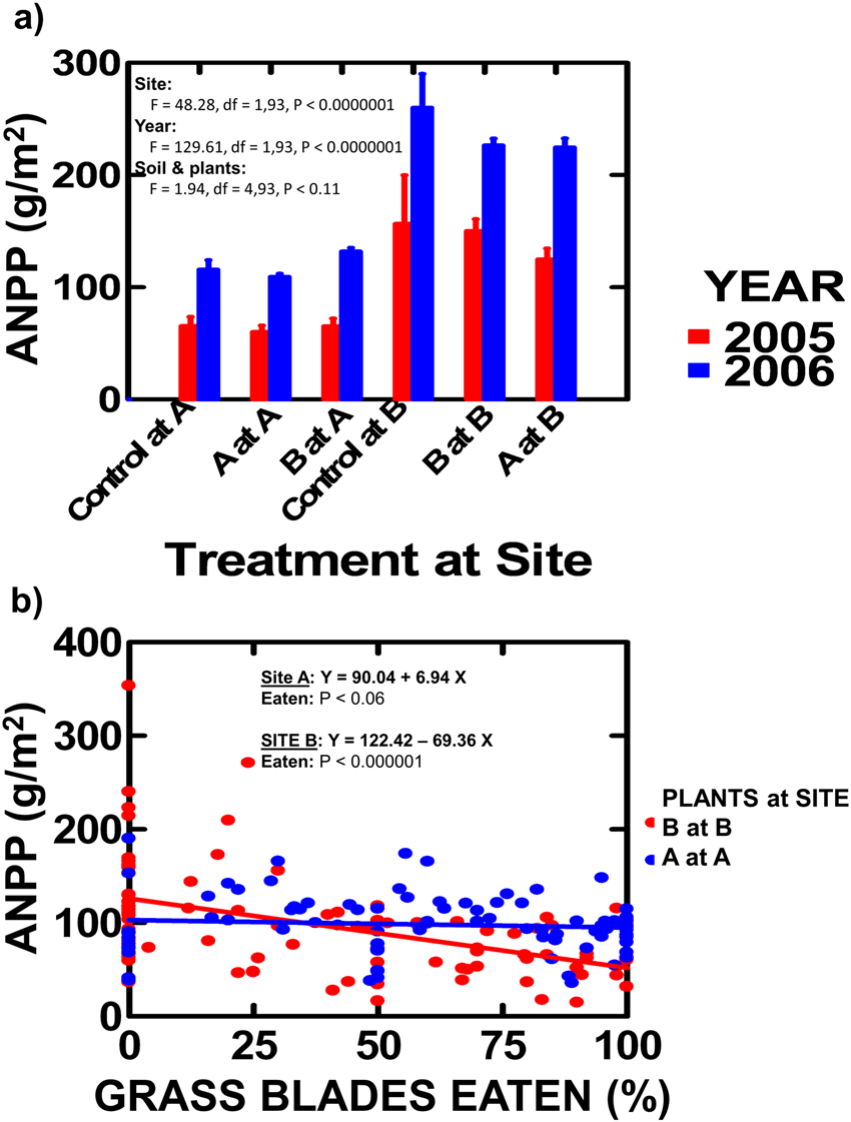
“Common garden” results. (**a**) Comparison of ANPP (±SE) measured without grasshoppers (2005–2006) for pots with Site A plants and soil, Site B plants and soil, and control plots at each site. ANOVA results are presented. (**b**) The relationship between ANPP versus proportion of grass blades eaten by grasshoppers in the prior year (2007–2013) at each site for control plots and pots with that sites’ plants. Results are presented separately as the slopes differ (P < 0.0002, see ANCOVA in Table S2.4).

ANPP was measured in June 2005 and 2006 (before adding grasshoppers). Several patterns emerged for ANPP when pots containing that site’s soil and plants, pots containing the other site’s soil and plants, and controls were compared (Fig. 1a):

1. Pots reflect field ANPP, as control areas at each site did not differ from pots with the site’s soil and plants (Tukey post hoc: P < 0.99 and 0.61 for A and B respectively).
2. Sites (B > A) and years (2006 > 2005) differed in ANPP (Table S2.1: given sample size, only 2-way interactions could be examined and none were significant), with a weaker soil source effect (B > A: A = heavy clay, B = diatomaceous clay/sand).

Abiotic effects are expected given site, year and soil responses (Table S2.1). Two common abiotic effects on ANPP are soil moisture and growing season N. Soil moisture results (Table S2.2) are Site A > B (Site A has more precipitation, being closer to mountains); 2006 > 2005 (prior precipitation was 32.7 cm in 2005 vs. 39.2 cm in 2006); B’s soil > A’s (diatomaceous clay/sand retains water better than heavy clay). If greater soil moisture increases ANPP, then site moisture is opposite ANPP observations, but year and soil type are consistent with ANPP observations. Growing season N results (Table S2.3) are Site B > A; 2005 > 2006; A’s soil > B’s. If greater N increases ANPP, then site N is consistent with ANPP observations, but year and soil type are opposite ANPP observations. However, separating moisture and N effects is difficult as moisture and N are negatively correlated (r = 0.34, n = 100, P < 0.0004). Regardless, strong abiotic effects on ANPP are evident.

Biotic effects in the absence of grasshoppers can only emerge with plant source, but no main or interaction (Tables S2.1, S2.2 and S2.3) effect approached significance (P never < 0.19) for ANPP, soil moisture or growing season N.

### With grasshoppers

ANPP was measured in June for 2007–2013. This reflects growth after prior, not current, year’s grasshopper consumption, as >90% of grasshoppers in the current year hatch after ANPP is attained. ANPP responses to prior year’s grasshopper feeding intensity (% grass blades fed on) were examined in pots with the plant source from that site and controls (Fig. 1b):

1. As without grasshoppers, ANPP with 0% feeding intensity (intercept) was greater for Site B than Site A plants (147.6 vs. 96.4 g/m^2^, t = 3.94, df = 93, P < 0.0002).
2. With grasshoppers, ANPP differs with plant source (slopes - Fig. 1b: F = 1.47; df = 1,176; P < 0.0002), as Site A plants now provide greater ANPP than Site B plants.
3. With grasshoppers, the soil effect on ANPP now disappears (Table S2.4).

Therefore, ANPP between sites was reversed with herbivory (Fig. 1a vs. b), challenging the traditional view that abiotic factors solely set ANPP.

Abiotic effects on ANPP, when grasshoppers were absent, appeared for soil moisture and growing season N (Tables S2.2 and S2.3). Soil moisture effects with grasshoppers were unchanged (Site A > B; Soil A < B; annual differences; no plant source effect: Table S2.5). Annual differences in N with grasshoppers still appear, but differences due to site or soil disappear, and now a significant effect of plant source appears. Therefore, two biotic effects, plant source and grasshoppers, affect N which is the limiting nutrient for plant growth.

**Figure 2:**
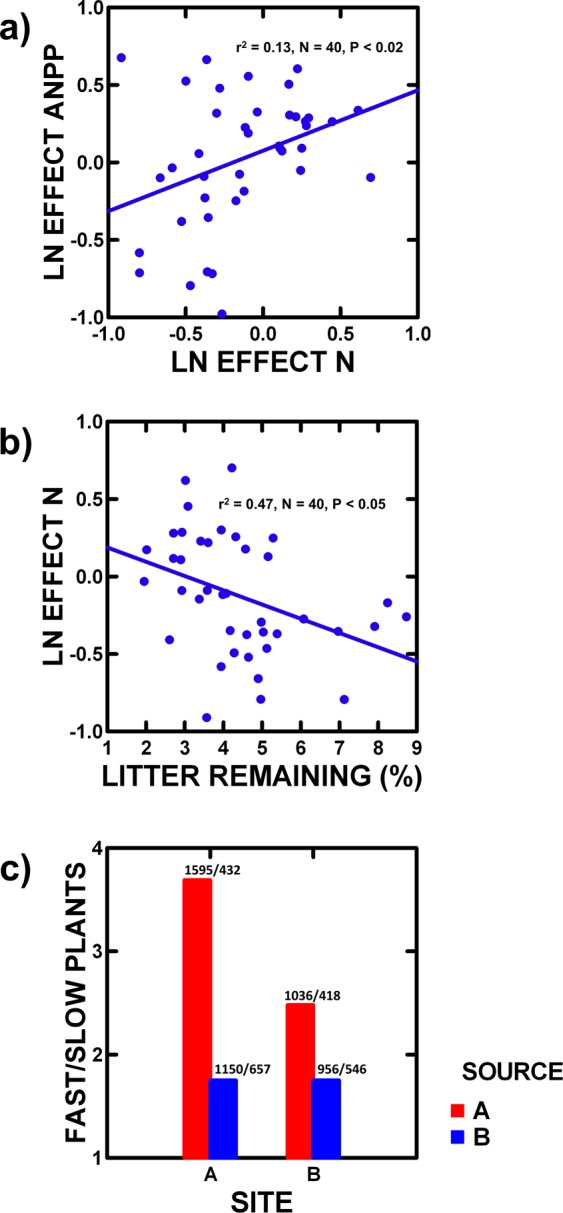
(**a**) The inter-annual change in ANPP (ANPP effect = current year ANPP/prior year ANPP) and the inter-annual change in growing season N (N effect = current year N/prior year N) in experimental pots, and regression statistics. (**b**) The inter-annual change in growing season N (N effect = current year N/prior year N) and the percentage of litter remaining (100% X litter in Sept/prior year ANPP) in experimental pots, and regression statistics. (**c**) Relative abundance of fast (*P. pratensis*) to slow (*P. smithii*) decomposing plants at each site with each plant source after grasshoppers were present in experimental pots. The number of plants sampled are also provided.

Biotic effects on ANPP emerge with plant source and past amount of grasshopper feeding (Table S2.4). As in our earlier studies^9–11^, ANPP with grasshoppers rises slightly for Site A’s plants at Site A (3%), but falls dramatically for Site B’s plants at Site B (41%), a 44% net change (Table S2.4). Soil moisture cannot account for this as it did not change with vs. without grasshoppers; however, N did change, and as in our prior observational studies^9–12^, inter-annual changes in ANPP and N are positively correlated (Fig. 2a). Without grasshoppers, Site B’s N was 37% greater than A’s, but with grasshoppers, it was 16% less, a 53% net change (Tables S2.3 and S2.6). With grasshoppers, plant source accounts for a portion of this change in N, as Site A plants increase N by 5% and Site B plants decrease N by 14%, a 19% net change. Grasshopper excrement and carcasses, even though highly facile, provide minimal N^9^, so that grasshoppers must affect N in additional ways.

One way that N availability can be biotically affected is through plant litter decomposition rate, as inter-annual change in N decreases as percent of litter remaining (litter in Sept/prior year ANPP) increases (Fig. 2b). With grasshoppers, percent of litter remaining was lower at Site A, especially for Site A plants at Site A (Table S2.7). This accounts for a 26% net change in N between Site A and B, as plant N content does not differ between sites^10,11^. Therefore, added to a 19% net change in N due to plant source, a total net change of 45% arises which compares to the observed 42%.

Without grasshoppers, the initial equal relative abundance of the two plants did not change (*P. pratensis* at Site A = 42.5%: χ^2^ = 2.40, P < 0.14; at Site B = 48.9%: χ^2^ = 0.02, P < 0.88), and the sites did not differ (χ^2^ = 0.53, P < 0.47). With grasshoppers, the faster decomposing *P. pratensis* increased in relative abundance at both sites, but much more for plants from Site A (77%: χ^2^ = 114.6, P < 0.000001). While the faster decomposing plant relative abundance did not differ between sites for plants from Site B (P < 0.99), it was 27% greater for Site A plants at A than B. Therefore, species composition of the litter changes with grasshoppers, which accounts for decomposition rate and N differences, as previously suggested^8–10^.

Litter plant species composition changes between the two sites due to grasshopper selective feeding, as grasshoppers feed on average 28% more on the slower decomposing plant at Site A than at B, and this preference is 55% greater for Site A plants. Therefore, grasshoppers increase faster decomposing plant relative abundance at Site A, especially for Site A plants, and thereby increase N availability and ANPP at Site A over B, as observed.

**Figure 3:**
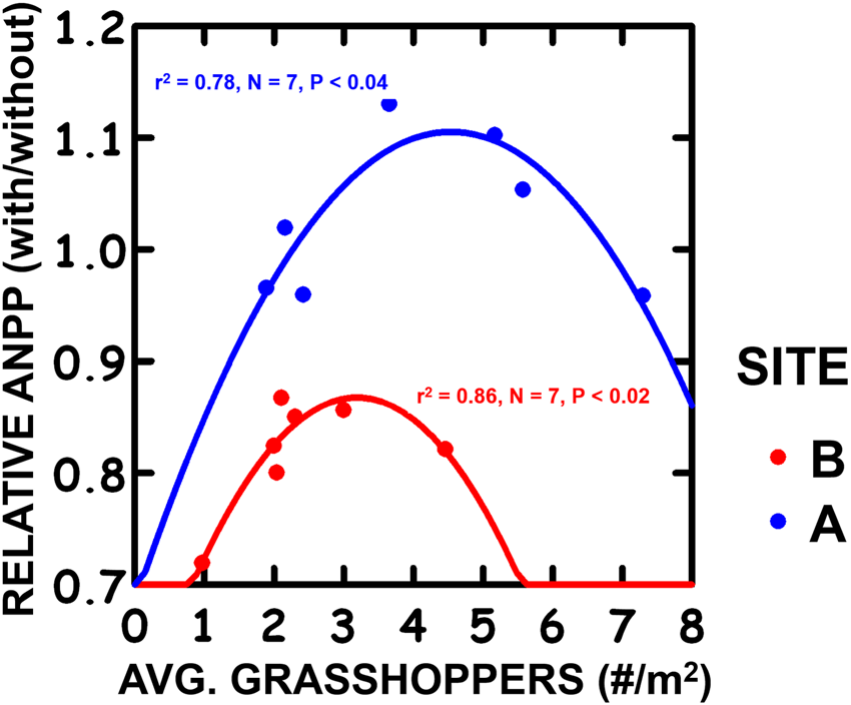
The ANPP response to grasshopper herbivory (ANPP with grasshoppers ÷ ANPP without grasshoppers) for plants from Site A at Site A and plants from Site B at Site B, and quadratic regression statistics.

A final biotic effect emerges with grasshoppers, as Site A plants do better at Site A than at Site B, and vice versa. This might be expected with local adaptation to the abiotic conditions of greater moisture and lower N at Site A than at Site B. While a response to abiotic conditions should be observed with or without grasshoppers (Tables S2.1 and S2.4), it was only observed with grasshoppers. This may reflect local adaptation to different levels of grasshopper herbivory. From 1995–2013, grasshopper densities at Site A were greater than at Site B (13.4/m^2^ vs. 6.1/m^2^, P < 0.01), so herbivory may be a stronger selection pressure at Site A. Local adaptation to herbivory can be examined by plotting relative ANPP (with ÷ without grasshoppers) vs. grasshopper density. The plot increases and then decreases as grasshopper density increases for both Site A and B plants (Fig. 3). However, Site B plant relative ANPP is always <1 (i.e., ANPP with grasshoppers always is less than without grasshoppers) and declines at low grasshopper densities, while Site A plant relative ANPP can be >1 (ANPP with grasshoppers can be greater than without grasshoppers) and declines at high grasshopper densities.

## Discussion

We conducted a “common garden” experiment at two sites over 10 years, which created different ecosystems by exchanging plants (same species) and soil between sites, while removing or allowing herbivory, to examine ecosystem functioning (N cycling and ANPP). While experimental ecosystems have been studied, e.g.^13–15^, we believe this to be the first *in situ* field experiment to examine the independent role of abiotic and biotic factors relative to the natural systems. The two sites reflect different microclimates: the 10 years of different weather, and the two site’s different soil moisture and nitrogen properties—abiotic conditions. The plants from each site and absence/presence of herbivory reflect different biotic food web conditions.

### Without grasshoppers

Without herbivory, as traditionally expected^1^, N cycling and ANPP were driven solely by abiotic conditions (site, year and soil) and the only available biotic factor, plant source, had no effect. One site (B) was 26% more productive due to having greater nitrogen availability, while inter-annual variability in productivity and soil effects were driven by moisture. N may limit ANPP more than moisture, as the more productive site (B) had greater N, but less moisture, than the other site (A). This is consistent with experimental supplementation of N at both sites, that found N to limit ANPP^8,9,11^. In summary, without herbivory (no grasshoppers), the traditional view that ANPP primarily is driven abiotically by microclimate (site), annual weather (year), and soil is supported.

### With grasshoppers

With herbivory (grasshoppers), the relative productivity of the two sites reversed; so the less productive site in the absence of herbivory had a 6% increase in ANPP but was now 15% more productive than the more productive site in the absence of herbivory, which had a 33% decline in ANPP. This occurred even though the more productive site in the absence of herbivory experienced 55% less herbivory (lower grasshopper density).

The reversal was due to changes in litter decomposition due to herbivory. Litter decomposition rate is biotically affected by the plant species comprising the litter. *P. pratensis* litter decomposes faster than *P. smithii*^9–11^ litter, so percent of litter remaining will decrease and N will increase as relative abundance of *P. pratensis* litter increases. Grasshopper selective feeding enhanced N at the originally less productive site (A) by increasing the relative abundance of faster decomposing plants (*P. pratensis*), and diminished N at the originally more productive site (B) by increasing the relative abundance of slower decomposing plants (*P. smithii*). Preferential feeding arises because grasshoppers prefer green vegetation that is lower in water content, so *P. pratensis* at Site B is preferred, while *P. smithii* is preferred at Site A^9–11^.

With herbivory, we also observed that the plant species from the different sites were locally adapted to herbivory, even though they are the same species, with the plants from the site (A) where the herbivore was consistently more abundant being able to cope better with herbivory. A number of field experiments have reported on the importance of local adaptation of plants to herbivory, e.g.^15–17^, and at least one study has hypothesized that this might affect nutrient dynamics^18^.

In summary, grasshoppers affect ANPP through plant source, plant species composition and level of herbivory. Counter to tradition that ANPP is driven by abiotic effects, biotic effects emerge stronger, increasing ANPP at one site and decreasing it at another beyond abiotic limits. This is driven by herbivory creating positive feedbacks that affect productivity, as reported elsewhere, e.g.^4–7,19–27^. While this did not negate abiotic influences, it surpassed their effect, as hypothesized earlier^3^.

### Implications

Our study indicates that focusing solely on the abiotic effects, and ignoring biotic influences, can be very misleading in understanding ecosystem functioning. This includes misrepresenting system productivity by as much as 33%, and misidentifying the most productive system. Furthermore, the biotic influences operate on a local spatial scale (few kilometers). Consequently, we suggest that characterizing ecosystem functioning based solely on large spatial scale abiotic conditions (e.g., precipitation and temperature), as commonly conducted, especially in making climate change projections, may be misleading. Furthermore, environmental changes often change species abundances, including lost and added species, so that novel biotic effects may arise^14,28,29^. This requires better and more detailed ecological insights^30^.

## Materials and Methods

### Materials

The “common garden” experimental ecosystems were created in June 2004. Forty large plastic pots (0.053 m^2^: diameter = 26 cm, depth = 30 cm) with the bottom cut out were buried (28 cm) in the ground. Holes closely fit a pot and the soil that was removed to make the hole was saved, maintaining its profile and the surface was labelled. Pots were installed 2 m apart in a 4 × 10 grid.

### Treatments

Pots were randomly assigned to 4 treatments (Site A plants – Site A soil; Site B plants – Site B soil; Site A plants - Site B soil; Site B plants - Site A soil) with 10 replicates for each. Each pot’s soil treatment was provided by soil saved from a hole cut for a pot at the appropriate site. Each pot’s plant treatment was provided by transplanting plants from the appropriate site. Based on abundance and plant size at the sites in June 2004, five 20 cm (±10%) tall individuals of each of two grass species (*Poa pratensis* and *Pascopyrum smithii*) were planted in a pot. Plants were carefully dug up to maintain root mass (~20 cm around plant) and soil was gently washed away. To help establish plants, 0.25 L of water/pot was provided weekly from June–Sept 2004 and any other plants were removed. To exclude grasshoppers, pots were covered by a nylon insect screen bag (0.5 m tall), which was supported by wire and attached to the pot with plastic clips.

Pots had grasshoppers excluded during 2004–2005 (late June–September). Starting in late June 2006, grasshoppers (*Melanoplus sanguinipes*) at that year’s field density were allowed to feed in half of the pots in each treatment. This was accomplished by placing the appropriate number of grasshoppers in a pot for 1–2 days every 2 weeks, where the number of grasshoppers and length of time the grasshoppers were allowed to feed in a pot was based upon their mean field density over the preceding 2 weeks (See Table S1 in Supplementary Information). The field density was determined from two (11 AM and 2 PM) weekly counts of grasshoppers in twenty-four 0.1 m^2^ rings^31^.

Five 0.11 m^2^ (0.33 m × 0.33 m) control areas were randomly placed immediately outside the grid of 40 pots at each site, but within the experimental area. Grasshoppers were excluded from each control in years 2004–2005, using a 0.5 m high box of aluminum insect screen supported by wooden stakes and attached to garden edging by folding^32^. From 2006–2012, the insect screen box was removed, so that grasshoppers had access.

### Measurements

The following measurements were made from 2005–2013 in the pots and controls (see Table S1):

1. Annual aboveground net primary production (ANPP) was measured nondestructively using a ©CropScan radiometer every two weeks from June–Oct^33,34^. The radiometer was positioned to measure a 0.05 m^2^ area. Live plant biomass was computed from radiometer readings using site and date specific regressions. Each regression was based on readings from 3–5 0.1 m^2^ plots adjacent to the grid of pots, which were selected to range from low to high plant abundances, and from the plant biomasses of these 3–5 0.1 m^2^ plots. Green vegetation in each of these plots was clipped, dried for 48 hours at 60 °C, and weighed. Regressions averaged an r of 0.93 (±0.03 SE; range: 0.74–0.99). ANPP is the sum of all increases in green plant biomass between consecutive periods from June–Oct; however, we only report the highest values for June as they always decreased afterwards.
2. Absolute and relative abundances of the two grasses (*P. pratensis* and *P. smithii*) were determined using point frame sampling^35^. Twenty-five random points in each pot and control were identified in June and Sept as: bare ground, litter, moss, or plant species. Plant abundances were examined using the point frame “hits” on each species.
3. Nitrogen availability (inorganic) was measured in each pot and control twice a year (June–Sept, Oct–May) with an ion exchange resin bag (©Rexyn) buried 15 cm in each pot and control in June and Oct^36^. Bags were kept frozen after collection until N was extracted with 2 M KCl, and measured as NH_4_^+^ and NO_3_^−^ with a ©Latchat spectrophotometer^37^. We only report June–Sept values, as these were the only values found to change in the experiment.
4. Soil moisture was measured every 2 weeks as conductivity using a gypsum block buried 20 cm in each pot and control^38^, which was correlated with soil moisture (% H_2_O).
5. Litter abundance in pots and controls was measured in Sept by removing it, drying it for 48 hours at 60 °C, weighing it and then returning it to its pot or control.
6. Percentage of grass blades eaten was measured in Oct by examining up to 25 blades of each of the two grass species for feeding damage by grasshoppers. No attempt was made to quantify the amount of tissue removed from a blade, just damage or no damage was recorded.

### Statistics

GLM, ANCOVA, regression and χ^2^ analyses were conducted using ©SYSTAT 13. Data employed in GLMs and ANCOVAs were normally distributed. Data from each individual pot with and without grasshoppers were employed in GLMs and ANCOVAs for ANPP, N and soil moisture (Table S2.1–6). Insufficient litter for each pot was measured in each year to perform statistics using each pot; therefore, litter values with grasshoppers were averaged over all years (N = 4 treatments X 2 sites X 5 years = 40) for the litter production GLM (Table S2.7) and regressions (Fig. 2a,b). Effect sizes (current ÷ prior measures) were natural log transformed (Fig. 2a,b). For GLMs and ANCOVAs, all main effects and statistically significant (P < 0.05) interaction terms were presented (Table S2). Fast vs. slow plant relative abundance was examined with χ^2^ analysis employing the sum of all point samples with grasshoppers over the five years in the treatments being compared. Finally, the ANPP responses for Site A and B plants to grasshopper density were analyzed by regression for the mean of all pots (Fig. 3).

## Supplementary information

Supplementary Information

## References

[1] Golley, F. B. *A History of the Ecosystem Concept in Ecology: More Than the Sum of the Parts*. (Yale University Press, 1996).

[2] Reeves MC, Moreno AL, Bagne KE, Running SW (2014). Estimating climate change effects on net primary production of rangelands in the United States. Climatic Change.

[3] Hutchinson GE, Deevey ES (1949). Ecological studies on populations. Biological Progress.

[4] McNaughton SJ, Ruess RW, Seagle SW (1988). Large mammals and process dynamics in African ecosystems. BioScience.

[5] Pastor J, Naiman RJ, Dewey B, McInnes P (1988). Moose, microbes, and the boreal forest. BioScience.

[6] Wardle, D. A. *Communities and Ecosystems: Linking the Aboveground and Belowground Components*. (Monographs in Population Biology, Princeton University Press, 2002).

[7] Bardgett RD, Wardle DA (2003). Herbivore-mediated linkages between aboveground and belowground communities. Ecology.

[8] Bardgett, R. D. & Wardle, D. A. *Aboveground-Belowground Linkages, Biotic Interactions, Ecosystem Processes, and Global Change*. (Oxford Series In Ecology and Evolution, Oxford University Press, 2010).

[9] Belovsky GE, Slade JB (2000). Insect herbivory accelerates nutrient cycling and increases plant production. PNAS.

[10] Belovsky GE, Slade JB (2002). An ecosystem perspective on grasshopper control: possible advantages to no treatment. JOR.

[11] Belovsky GE, Slade JB (2018). Grasshoppers affect grassland ecosystem functioning: Spatial and temporal variation. Basic Appl. Ecol..

[12] Belovsky, G. E. Do grasshoppers diminish productivity? A new perspective for control based on conservation in *Grasshoppers and Grassland Health: Managing Grasshopper Outbreaks Without Risking Environmental Disaster* (eds Lockwood, J. A., Latchinsky, A. V. & Sergeev, M. G.) 7–29 (Kluwer Academic, 2000).

[13] Lawton JH (1995). Ecological experiments with model systems. Science.

[14] Loreau M (2001). Biodiversity and ecosystem functioning: current knowledge and future challenges. Science.

[15] Cook-Patton SC, McArt SH, Parachnowicz A, Thaler JS, Agrawal AA (2011). A direct comparison of the ecosystem and community impacts of genotypic and species diversity. Ecology.

[16] Agrawal AA, Hastings AP, Johnson MT, Maron JL, Salminen JP (2012). Insect herbivores drive real-time ecological and evolutionary change in plant populations. Science.

[17] Han PG, Agrawal AA, Sussman KI, Maron JL (2109). Population variation, environmental gradients and the evolutionary ecology of plant defense against herbivory. American Naturalist.

[18] Burghardt KT, Bradford MA, Schmitz OJ (2018). Acceleration and deceleration of litter decomposition by herbivory depends on nutrient availability through intraspecific differences in induced plant resistance traits. Journal of Ecology.

[19] Morrien E, Engelkes T, van der Putten WH (2011). Additive effects of aboveground polyphagous herbivores and soil feedback in native and range expanding exotic plants. Ecology.

[20] McNeil S, Cushman J (2005). Indirect effects of deer herbivory on local nitrogen availability in a coastal dune ecosystem. Oikos.

[21] Lovett G, Ruesink A (1996). Carbon and nitrogen mineralization from decomposing gypsy moth frass. Oecologia.

[22] Gruner D (2008). A cross-system synthesis of consumer and nutrient resource control on producer biomass. Ecol. Lett..

[23] Chapman S, Hart H, Cobb N, Whitham T, Koch G (2003). Insect herbivory increases litter quality and decomposition: an extension of the acceleration hypothesis. Ecology.

[24] Pringle R, Doak D, Brody A, Jocqué R, Palmer T (2010). Spatial pattern enhances ecosystem functioning in an African savanna. PLOS Biol..

[25] Hunter MD (2001). Insect population dynamics meets ecosystem ecology: effects of herbivory on soil nutrient dynamics. Agric. Forest Entomol..

[26] Burkepile DE, Hay ME (2008). Herbivore species richness and feeding complementarity affect community structure and function on a coral reef. PNAS.

[27] Pringle RM, Young TP, Rubenstein DI, McCauley DJ (2007). Herbivore-initiated interaction cascades and their modulation by productivity in an African savanna. PNAS.

[28] Crowl TA, Crist TO, Parmenter RR, Belovsky GE, Lugo AE (2008). The spread of invasive species and infectious disease as drivers of ecosystem change. Front. Ecol. Environ..

[29] Belovsky GE (1994). Management of small populations: concepts affecting the recovery of endangered species. Wildlife Soc. B..

[30] Belovsky GE (2004). Ten suggestions to strengthen the science of ecology. BioScience.

[31] Onsager JA, Henry JE (1977). A method for estimating the density of rangeland grasshoppers (Orthoptera: Acrididae) in experimental plots. Acrida.

[32] Belovsky GE, Slade JB (1995). Dynamics of two Montana grasshopper populations: relationships among weather, food abundance and intraspecific competition. Oecologia.

[33] Pearson RL, Miller LD, Tucker CJ (1976). Handheld spectral radiometer to estimate gramineous biomass. Appl. Optics.

[34] Milton EJ (1987). Principles of field spectroscopy. Int. J. Remote Sens..

[35] Daubenmire, R. F. Steppe Vegetation of Washington. (Washington Agricultural Experiment Station, Technical Bulletin 62, 1970).

[36] Binkley D, Hart SC (1989). The components of nitrogen availability assessments in forest soils. Adv. Soil Sci..

[37] Robertson, G. P. *et al*. (eds). Standard Soil Methods for Long-Term Ecological Research. (Oxford University Press, 1999).

[38] Dobriyal P, Qureshi A, Badola R, Hussain SA (2012). A review of methods available for estimating soil moisture and its implications for resource management. J. Hydrol..

