# Supplementary material for: Biotic Versus Abiotic Control of Primary Production Identified in a Common Garden Experiment
Source: Sci Rep. 2019 Aug 19;9:11961. doi: 10.1038/s41598-019-48512-7 (PMC6700119; doi:10.1038/s41598-019-48512-7)
Supplement: Supplementary file 1 — Supplementary Information [file 41598_2019_48512_MOESM1_ESM.pdf]

Supplementary Information:

Title: Biotic Versus Abiotic Control of Primary Production Identified in a Common Garden Experiment

Authors: Gary E. Belovsky and Jennifer B. Slade

TABLE S1. Average measurements (+ SE).

			SITE A						SITE B					
YEAR	GRASSHOPPERS	SITE SOURCE of SOIL/PLANT	ANPP (Jun) (g/m^2)	MOISTURE (Jun-Sep) (% mass)	N (Jun-Sep) (mg/bag)	LITTER (Sep) (g/pot)	HOPPERS (prior yr Jun-Sep) (avg. #)	EATEN (Oct) (%)	ANPP (Jun) (g/m^2)	MOISTURE (Jun-Sep) (% mass)	N (Jun-Sep) (mg/bag)	LITTER (Sep) (g/pot)	HOPPERS (prior yr Jun-Sep) (avg. #)	EATEN (Oct) (%)
2005														
CONTROL	NO		65.34±8.27	4.7±1.3	11.31±2.10				156.44±43.37	4.1±0.7	3.82±1.58			
POTS	NO	A/A	60.05±5.78	5.4±2.2	5.16±0.37				124.78± 9.73	4.9±1.5	9.64±0.98			
		A/B	43.94±6.81		4.21±0.65				107.91±11.65	4.0±0.3	10.99±1.15			
		B/A	68.19±7.13	5.4±0.4	3.96±0.60				140.09±11.86	4.9±2.2	7.81±3.66			
		B/B	65.20±6.88	5.4±1.3	2.81±0.42				149.99±10.67	4.6±1.2	6.30±0.84			
2006														
CONTROL	NO		117.05±7.66	6.3±4.5	18.14±4.12			***	259.65±13.6	4.8±1.5	1.79±0.93			***
POTS	NO	A/A	130.56±6.11	6.2±7.9	4.57±1.34			***	224.68±8.22	5.4±1.5	3.72±0.43			***
		A/B	119.75±7.38	6.9±2.6	2.44±0.18			***	219.89±3.64	5.2±2.1	3.92±0.37			***
		B/A	117.22±6.08	6.2±4.8	3.08±0.51			***	232.00±7.31	5.4±0.5	3.06±1.08			***
		B/B	109.16±5.86	7.2±1.2	2.75±0.41			***	226.27±6.20	5.4±0.9	1.95±0.36		**	***
2007														
CONTROL	YES		151.77±13.0	5.3±3.4	5.51±1.41	4.31±1.41	2.05	38.7±18.7	133.42±12.8	4.3±1.0	1.52±0.34	5.87±1.54	1.90	0±0
POTS	YES	A/A	144.29±5.81	6.4±9.6	1.67±0.42	4.60±0.40	2.05	76.7±6.8	49.20±2.70	5.7±3.3	2.34±1.31	3.82±0.53	1.90	70.9±3.4
		A/B	130.72±10.3	6.6±6.7	1.49±0.41	4.52±0.43	2.05	72.6±5.5	51.85±4.86	5.6±4.1	1.09±0.12	3.39±0.39	1.90	67.8±6.6
		B/A	147.07±8.80	6.3±14.	1.59±0.24	4.56±0.48	2.05	63.5±5.4	50.36±5.37	6.3±5.4	2.13±1.02	3.78±.040	1.90	67.0±7.7
		B/B	137.65±7.04	6.8±3.2	1.16±0.33	4.36±0.54	2.05	77.8±8.1	57.17±3.81	6.5±3.7	0.69±0.31	3.97±0.66	1.90	79.3±7.7
2008														
CONTROL	YES		65.63±22.3	7.1±3.8	7.37±1.66	5.42±1.80	2.11	81.8±8.7	69.02±3.84	4.9±2.0	4.30±0.92	3.10±0.47	3.66	3.6±3.6
POTS	YES	A/A	98.28±7.52	6.9±1.6	2.82±0.57	4.58±0.51	2.11	55.9±7.5	95.19±8.30	6.0±6.7	3.36±0.80	4.80±0.88	3.66	55.0±4.8
		A/B	64.34±8.60	6.9±5.2	2.13±0.29	4.49±0.38	2.11	52.4±7.6	87.39±8.45	5.1±1.9	1.79±0.26	3.75±0.67	3.66	60.5±6.2
		B/A	102.27±6.83	6.7±3.4	2.26±0.30	4.30±0.42	2.11	45.1±8.0	81.00±5.06	7.5±2.9	2.81±1.71	4.93±0.96	3.66	42.1±7.7
		B/B	67.30±8.18	7.9±2.1	2.57±0.41	3.35±0.32	2.11	55.7±8.5	112.03±8.79	7.8±1.8	1.73±0.44	4.01±0.43	3.66	49.4±9.3
2009														
CONTROL	YES		88.85±17.0	8.2±2.0	13.32±3.19	3.18±0.45	3.01	20.0±12.2	140.95±11.80	10.1±0.3	4.29±0.78	2.75±0.44	5.18	20.0±20.1
POTS	YES	A/A	107.38±8.19	10.1±0.8	2.19±0.65	4.65±0.66	3.01	89.1±9.8	35.69±7.31	10.6±0.2	4.37±0.97	3.12±0.97	5.18	86.7±5.5
		A/B	87.06±6.00	10.9±0.7	1.79±0.49	3.99±0.49	3.01	81.6±7.1	48.63±7.96	10.7±0.3	3.97±0.97	3.47±0.55	5.18	90.6±2.5
		B/A	93.57±4.69	9.8±1.3	3.30±1.07	4.32±0.58	3.01	86.5±6.4	39.39±4.75	11.1±0.4	3.89±1.27	3.12±0.50	5.18	83.2±9.9
		B/B	92.18±8.11	11.2±0.8	3.46±0.76	4.60±0.93	3.01	92.1±5.1	50.45±7.03	11.0±0.4	2.76±0.39	2.59±0.33	5.18	73.4±10.4
2010														
CONTROL	YES		79.53±4.44	10.5±1.3	6.89±1.90	1.40±0.33	2.31	33.3±19.0	80.24±13.0	6.9±7.5	3.48±0.54	2.85±0.45	5.58	0±0
POTS	YES	A/A	85.19±4.03	10.8±1.4	3.18±0.73	4.60±1.09	2.31	93.3±5.4	65.10±10.1	10.1±0.7	3.48±1.08	2.23±0.19	5.58	80.3±6.5
		A/B	78.59±4.31	11.5±2.0	3.47±0.73	3.86±0.70	2.31	88.0±6.5	63.19±8.84	9.3±2.5	3.01±0.47	1.72±0.19	5.58	86.8±6.6
		B/A	86.46±2.92	11.0±0.7	3.83±0.48	2.93±0.48	2.31	81.5±7.3	52.34±9.09	10.9±0.8	2.89±0.73	2.07±0.47	5.58	65.5±8.6
		B/B	76.34±4.04	10.6±2.0	3.90±1.33	3.05±0.52	2.31	93.6±5.0	83.31±8.79	11.4±1.2	2.33±0.41	1.69±0.32	5.58	79.0±4.4
2011														
CONTROL	YES		99.54±5.61	8.1±7.1	4.53±1.62	3.08±0.55	4.47	33.2±9.4	149.27±24.2	4.9±2.0	1.14±0.11	4.65±0.52	7.30	15.2±3.6
POTS	YES	A/A	92.96±6.32	6.4±4.9	2.84±0.82	2.53±0.29	4.47	49.0±13.2	80.19±13.4	6.8±4.3	2.11±0.49	3.61±0.43	7.30	40.6±6.5
		A/B	94.65±5.12	7.2±8.4	3.81±1.07	2.78±0.43	4.47	46.7±6.4	79.85±13.0	6.0±12.	2.27±0.61	2.35±0.24	7.30	47.8±4.3
		B/A	91.23±3.94	6.5±2.7	3.94±1.13	2.39±0.32	4.47	50.0±7.0	90.94±9.11	7.5±1.7	3.17±0.73	3.27±0.59	7.30	40.6±2.8
		B/B	84.56±6.06	6.8±5.0	3.51±0.71	2.46±0.45	4.47	65.2±6.5	69.87±6.57	7.3±1.8	3.53±0.75	3.17±0.45	7.30	52.6±7.9
2012														
CONTROL	YES		137.90±13.0	8.9±2.0	4.11±0.57	***	2.01	0±0	149.77±38.77	4.1±1.0	3.70±0.89	5.83±0.52	2.43	0±0
POTS	YES	A/A	128.29±7.95	7.1±0.8	2.83±0.46	4.09±0.75	2.01	62.2±10.0	54.40±18.62	4.7±0.5	2.99±0.40	4.49±0.76	2.43	5.0±5.0
		A/B	122.74±5.43	8.1±4.8	2.43±0.22	3.80±0.53	2.01	56.0±5.7	99.72±13.67	4.5±0.6	2.54±0.31	4.07±0.43	2.43	4.6±3.1
		B/A	127.36±8.21	7.0±1.7	3.12±0.43	3.86±0.71	2.01	54.2±6.7	86.10±12.06	4.9±1.2	2.48±0.35	4.56±0.60	2.43	2.5±2.5
		B/B	113.14±11.0	7.3±3.1	2.94±0.51	2.52±0.36	2.01	60.3±7.0	72.56±15.06	5.4±1.0	2.08±0.22	3.07±0.47	2.43	11.3±7.2
2013														
CONTROL	YES		62.08±36.70				0.98		225.51±35.51				2.17	
POTS	YES	A/A	68.28±14.00				0.98		75.41±13.37				2.17	
		A/B	52.04±12.24				0.98		90.37±11.90				2.17	
		B/A	60.73±10.36				0.98		75.87±23.23				2.17	
		B/B	50.19±13.11				0.98		83.93±15.26				2.17	

*** not applicable as previously not protected from grasshoppers; ** average of all pots; *** not sampled**

**** average of all pots**

***** not sampled**

Supplementary Information:

Title: Biotic Versus Abiotic Control of Primary Production Identified in a Common Garden Experiment

Authors: Gary E. Belovsky and Jennifer B. Slade

Table S2. Statistical analyses.

WITHOUT GRASSHOPPERS:

	1) ANPP			2) SOIL MOISTURE			3) NITROGEN		
	GLM			GLM			GLM		
	F	df	P	F	df	P	F	df	P
**YEAR**	282.61	1,175	0.000001	29.96	1,95	0.000001	28.03	1,151	0.000001
**SITE**	383.61	1,175	0.000001	65.06	1,95	0.000001	13.54	1,151	0.0003
**SOIL**	6.6	1,175	0.01	7.63	1,95	0.006	7.43	1,151	0.007
**PLANTS**	1.05	1,175	0.31	0.11	1,95	0.74	1.70	1,151	0.19

WITH GRASSHOPPERS:

	4) ANPP			5) SOIL MOISTURE			6) NITROGEN			7) LITTER		
	ANCOVA			GLM			GLM			GLM		
**SITE A PLANTS**	r=0.79, N = 270											
	F	df	P	F	df	P	F	df	P	F	df	P
**YEAR**	63.89	5,256	0.000001	165.17	5,471	0.000001	7.19	5,470	0.000002	4.9	5,39	0.001
**SITE**	7.10	1,256	0.008	17.89	1,471	0.00002	0.13	1,470	0.72	11.51	1,39	0.0004
**SOIL**	2.08	1,256	0.15	12.63	1,471	0.0004	0.29	1,470	0.59			
**PLANTS**				2.15	1,471	0.14	4.82	1,470	0.02	2.19	1,39	0.14
**SITE X YEAR**	14.83	5,256	0.000001									
**PLANTS X SITE**										8.8	1,39	0.005
**% EATEN**	3.47	1,256	0.06									

**SITE B PLANTS**	r = 0.81, N = 270		
	F	df	P
**YEAR**	68.52	5,256	0.000001
**SITE**	41.83	1,256	0.000001
**SOIL**	3.48	1,256	0.07
**PLANTS**			
**SITE X YEAR**	7.54	5,256	0.000001
**% EATEN**	18.62	1,256	0.000001
